# Supplementary material for: Conjugative RP4 Plasmid-Mediated Transfer of Antibiotic Resistance Genes to Commensal and Multidrug-Resistant Enteric Bacteria In Vitro
Source: Microorganisms. 2023 Jan 12;11(1):193. doi: 10.3390/microorganisms11010193 (PMC9860721; doi:10.3390/microorganisms11010193)
Supplement: Supplementary file 1 [file microorganisms-11-00193-s001.zip › microorganisms-2107232-supplementary.pdf]

Table S1. Virulence genes present in Commensal gut *E. coli* LM715-1.

Gene Function	Gene
TonB-dependent hemin, ferrichrome receptor	*chuA*
Hemin transport protein HmuS	*chuS*
Periplasmic hemin-binding protein	*chuT*
Hemin ABC transporter, permease protein	*chuU*
ABC-type hemin transport system, ATPase component	*chuV*
Putative oxygen independent coproporphyrinogen III oxidase, heme uptake	*chuW*
Putative heme iron utilization protein	*chuX*
Flavin reductase	*chuY*
curli production assembly/transport protein CsgG	*csgG*
2,3-dihydro-2,3-dihydroxybenzoate dehydrogenase	*entA*
Enterobactin synthase component B	*entB*
Isochorismate synthase EntC	*entC*
Enterobactin synthase component D, phosphopantetheinyl transferase	*entD*
Enterobactin synthase component E	*entE*
Enterobactin synthase component F	*entF*
Enterobactin exporter EntS, iron-regulated	*entS*
Putative adhesin FdeC	*fdeC*
TonB-dependent receptor; Outer membrane receptor for ferric enterobactin and colicins B, D	*fepA*
Ferric enterobactin-binding periplasmic protein FepB	*fepB*
Ferric enterobactin transport ATP-binding protein FepC	*fepC*
Ferric enterobactin transport system permease protein FepD	*fepD*
Ferric enterobactin transport system permease protein FepG	*fepG*
Enterobactin esterase	*fes*
Type-1 fimbrial protein, A chain precursor	*fimA*
Type 1 fimbriae regulatory protein FimB	*fimB*
Chaperone protein FimC	*fimC*
Outer membrane usher protein FimD	*fimD*
Type 1 fimbriae regulatory protein FimE	*fimE*
Protein FimF (regulates length and adhesion of type 1 fimbriae)	*fimF*
Protein FimG (regulates length and adhesion of type 1 fimbriae)	*fimG*
Protein FimH (regulates length and adhesion of type 1 fimbriae, and mediates mannose binding)	*fimH*
Fimbrin-like protein FimI	*fimI*
Formate efflux transporter FocA	*focA*
Chaperone protein FocC	*focC*
Outer membrane usher protein FimD	*focD*
F1C Putative fimbrial adhesin precursor	*focH*
pesticin/yersiniabactin receptor protein	*fyuA*
General secretion pathway protein C	*gspC*
General secretion pathway protein D	*gspD*
General secretion pathway protein E	*gspE*
General secretion pathway protein F	*gspF*
General secretion pathway protein G	*gspG*
General secretion pathway protein H	*gspH*
General secretion pathway protein I	*gspI*
General secretion pathway protein J	*gspJ*
General secretion pathway protein K	*gspK*
General secretion pathway protein L	*gspL*
General secretion pathway protein M	*gspM*

Glycosyltransferase IroB	*iroB*
ABC transporter protein IroC	*iroC*
Trilactone hydrolase IroD	*iroD*
Periplasmic esterase IroE	*iroE*
Outer Membrane Siderophore Receptor IroN	*iroN*
iron acquisition yersiniabactin synthesis enzyme (irp1, polyketide synthetase)	*irp1*
yersiniabactin biosynthetic protein Irp2	*irp2*
Capsular polysaccharide export system periplasmic protein KpsD	*kpsD*
Capsular polysaccharide ABC transporter, permease protein KpsM	*kpsM*
Capsular polysaccharide ABC transporter, ATP-binding protein KpsT	*kpsT*
outer membrane protein A	*ompA*
Pap fimbrial major pilin protein	*papA*
Outer membrane usher protein PapC	*papC*
Chaperone protein PapD	*papD*
Fimbrial adapter PapF	*papF*
PAP fimbrial minor pilin protein	*papH*
regulatory protein PapI	*papI*
Per-activated serine protease autotransporter enterotoxin EspC / autotransporter domain, T5aSS type secretion	*pic*
Putative F1C and S fimbrial switch Regulatory protein	*sfaB*
S fimbrial switch regulatory protein SfaC	*sfaC*
S fimbriae minor subunit SfaD	*sfaD*
S fimbriae outer membrane usher SfaF	*sfaF*
S fimbriae minor subunit SfaG	*sfaG*
regulatory protein SfaX	*sfaX*
regulatory protein SfaY	*sfaY*
outer membrane heme/hemoglobin receptor ShuA	*shuA*
Tir domain containing protein TcpC	*tcpC*
Hemoglobin protease	*vat*
E. coli common pilus chaperone EcpE	*yagV/ecpE*
iron acquisition regulator (YbtA,AraC-like, required for transcription of FyuA/psn,Irp2)	*ybtA*
yersiniabactin siderophore biosynthetic enzyme, 2,3-dihydroxybenzoate-AMP ligase	*ybtE*
Iron siderophore ABC transporter, permease/ATP-binding protein YbtP	*ybtP*
Iron siderophore ABC transporter, permease/ATP-binding protein YbtQ	*ybtQ*
salicylate synthase Irp9 of siderophore biosynthesis	*ybtS*
Iron acquisition yersiniabactin synthesis enzyme YbtT	*ybtT*
Yersiniabactin synthetase, thiazolinyl reductase component Irp3	*ybtU*
putative signal transducer	*ybtX*
regulator protein EcpR	*ykgK/ecpR*
